# Editorial: Oral diseases and prevention in pregnant women, infants and preschool children

**DOI:** 10.3389/froh.2025.1764572

**Published:** 2026-01-09

**Authors:** Jieyi Chen, Sherry Shiqian Gao, Amit Arora

**Affiliations:** 1 Guanghua School of Stomatology, Hospital of Stomatology, Sun Yat-sen University, Gaungzhou, China; 2 Department of Stomatology, School of Medicine, Xiamen University, Xiamen, China; 3 Discipline of Public Health, School of Medicine, Faculty of Health, Western Sydney University, Penrith, NSW, Australia

**Keywords:** child, oral health, policy, prevention, risk factors, woman

The period from pregnancy through early childhood is a cornerstone for lifelong health (1). Oral health is a critical, yet often overlooked, component of this foundation. Gestational physiological changes during pregnancy can aggravate maternal oral diseases, influencing both birth outcomes and the child's future oral health (2). The early postnatal years are equally critical for shaping long-term oral health. During this period, the oral microbiome is assembled, primary teeth erupt, and dietary as well as oral health-related habits are formed.

This Research Topic brings together diverse research to advance our understanding and response to oral diseases in these vulnerable groups. The contributing articles can be synthesized into three themes: the burden and risk factors of early childhood caries (ECC), the biological and prenatal factors on oral health and the implementation of strategies for prevention and policy.

## The burden and risk factors of ECC

A clear understanding of the burden of ECC is the first step toward effective action. The study on the Global, Regional and National Burden of Deciduous Dental Caries (1990–2021) provided a critical macro-level perspective, revealing that despite modest global declines, ECC remained a global public health challenge. It disproportionately affected regions with medium and low socio-demographic indices. This report sounds an unequivocal warning that without targeted interventions, ECC will likely exacerbate existing health disparities.

Complementing this global view, several studies delved into the specific risk and protective factors at the individual level. Cross-sectional studies from Shijiazhuang and Huizhou, China, indicated a high prevalence of ECC in China and identified several key modifiable factors. High-frequency consumption of sugary foods and drinks before bed were significant related to the prevalence of ECC. In contrast, protective factors such as toothbrushing before sleep, the use of fluoride toothpaste and regular dental examinations were associated with reduced dental caries. Furthermore, the Shijiazhuang study moved beyond the oral cavity. It demonstrated a significant inverse correlation between dental caries severity and weight-for-height scores, underscoring the link between ECC and a child's nutritional status.

## Biological mechanisms and prenatal factors

The second theme explored in this Topic shifted the focus to the underlying biological mechanisms and the surprising prenatal factors that related to oral health. The comprehensive review on the Oral Microbiome in Children reframed our understanding of the oral cavity as a complex ecosystem. It detailed how modern genomic technologies had illuminated the shift from a symbiotic microbial community to a dysbiotic state that drive diseases like dental caries, providing the biological rationale for the behavioral risks identified elsewhere.

Adding a profound dimension to this study, the prospective birth cohort study on prenatal maternal hormones revealed that the blueprint for oral health may be laid before birth. It reported that higher maternal salivary levels of cortisol, estradiol and other hormones in late pregnancy were associated with the pace of primary tooth eruption in early childhood. The results suggested that prenatal milieu was an under-recognized determinant of dental development. This opens new research frontiers focused on how very early-life exposures influence lifelong oral-health outcomes.

## Implementing prevention: From education to policy

Knowing the “what” and “why” of ECC is futile without effective implementation of the “how.” The final group of articles addresses this translation gap, offering insights from the frontline of prevention. The systematic review on Carer Perspectives provided an essential human-centered lens, revealing that parents view oral health and weight holistically as part of overall “child wellness.” This finding is crucial for designing non-stigmatizing, strength-based health messages that resonate with families' lived experiences.

At the institutional level, the large-scale evaluation of an Oral Health Education program for Kindergarten Teachers in Hong Kong demonstrated a highly effective model for scaling prevention. The results showed that training teachers not only increased their knowledge and confidence but was also widely accepted, positioning educators as powerful multipliers of oral health promotion.

Finally, two articles challenge us to consider the systemic and legal frameworks necessary for protection and equity. The qualitative study on Audit and Feedback in Alaska Native healthcare settings showed that with adequate resources and a positive organizational culture, evidence-based practices could be successfully implemented even in challenging contexts, offering a model for reducing stark health inequities. On a broader scale, the compendium on Child Dental Neglect and Legal Protections across 26 countries revealed a critical policy-practice gap. It argued that while general child neglect laws were common, specific legislation on dental neglect was rare but essential for building a “supportive social ecosystem” that facilitates children's access to care.

## Conclusion: A synergistic path forward

The studies in this Topic argued that improving oral health in pregnant women and young children required a synergistic, multi-level approach. There was no single solution. Progress depended on integrating global surveillance with local evidence, biological insight with an understanding of human behavior, and clinical interventions with empowered communities as well as supportive policies. By viewing oral health not as an isolated concern but as an integral part of maternal and child well-being, we can forge a path toward a future where every child has the foundation for a healthy life, beginning with a healthy mouth.
